# Radiographic Classification of Hallux Valgus Interphalangeus Based on the Center of Rotation of Angulation (CORA): Prevalence, Reliability, and Surgical Implications

**DOI:** 10.7759/cureus.95752

**Published:** 2025-10-30

**Authors:** Suvank Rout, Souvagya Rout, Ronish Patidar, Timothy H Williams

**Affiliations:** 1 College of Medicine, Topiwala National Medical College and Bai Yamunabai Laxman Nair Charitable Hospital, Mumbai, IND; 2 Trauma and Orthopedics, Colchester General Hospital, Colchester, GBR; 3 Trauma and Orthopedics, Royal College of Surgeons of England, London, GBR; 4 Trauma and Orthopedics, First Faculty of Medicine, Charles University, Prague, CZE; 5 Orthopedics, Indira Gandhi Medical College, Shimla, IND

**Keywords:** ankle and foot, bone deformity correction, bunion correction, hallux disorders, hallux surgery

## Abstract

Introduction: Hallux interphalangeus (also called hallux valgus interphalangeus (HVI)) is a deformity characterized by lateral deviation of the distal phalanx of the great toe relative to the proximal phalanx. It is often seen in conjunction with hallux valgus but can occur in isolation. It is classically measured by the hallux interphalangeal angle (HIA), which is formed from the longitudinal axis bisections of the proximal and distal phalanges.

Aim: To recognize, discuss, and propose a classification for HVI considering the concept of center of rotation of angulation (CORA).

Methodology: We performed a retrospective analysis of pedobarographs performed at a single institution from December 2019 to December 2021. Inclusion criteria consisted of anterior-posterior view of foot X-rays of patients aged 18 years or older (skeletally mature). All radiographs had been performed with weight bearing. Excluded were those radiographs where a sagittal plane (flexion) deformity was seen on the lateral radiograph to reduce confounding factors. A total of 80 radiographs were collected from the patients included in this study. Results: The radiographical evidence showed that the prevalence of “normal” hallux in the patient cohort was 27.69% (n=18). The percentage of feet with HVI was 72.30% (n=47). Within the population who presented with HVI, it was noted that the commonest deformity, with 30.76% (n=20), presented with the CORA at the distal end of the proximal phalanx (P3). The second most prevalent type of HVI is with the CORA at the midpoint of the proximal phalanx (P2) (15.3%) (n=10). The remaining classes had a CORA based at the proximal end of the proximal phalanx (P1) at 10.7% (n=7), a CORA at the base of the distal phalanx (P4) (9.23%) (n=6), or a CORA within the distal interphalangeal joint (P5) (6.15%) (n=4).

Conclusions: Hallux interphalangeus is a clinically significant deformity that often coexists with or mimics hallux valgus. This study introduces a novel classification for HVI based on the CORA. We identified five deformity types and their distribution. This CORA-based classification provides a new anatomical framework for HVI, which may refine surgical planning beyond standard osteotomy techniques. Thus, a thorough understanding of the deformity’s etiology, radiographic assessment, and individualized treatment planning plays an essential role in achieving favorable outcomes.

## Introduction

Hallux valgus interphalangeus (HVI) is a deformity of the great toe characterized by an abnormal (usually increased) valgus angulation between the two phalanges. This has been historically ascribed to the wearing of shoes, but with later studies, it has been determined not only in adults and children but also in stillborn fetuses of several races, as noted by CH Barnett [1]. 

Pathological hallux interphalangeus is excessive clinodactyly of the great toe. It is classically measured by the hallux interphalangeal angle (HIA), which is formed from the longitudinal axis bisections of the proximal and distal phalanges. It is a common finding in the human population and can be seen both at birth and through the pediatric and adult populations.

In the radiological review of patients with normal and hallux valgus feet, Strydom et al. found an HVI prevalence of 62.1%, and a study by Barnett et al. found a prevalence of 82% in normal feet [1,2]. It is considered present if the HIA is greater than 10 degrees. An HIA >10° is generally considered abnormal, with values above ~14-15° carrying clinical significance [2,3].

The corrective surgical management of HVI is a well-recognized procedure, but to date utilizes a single concept correction through basal osteotomy, except for arthritic conditions [2]. As authors, we recognized patterns in these deformities reflecting nuances of the center of rotation of angulation (CORA) that point to probable background causes. Increasing our understanding of this can potentially influence surgical decision-making and optimize outcomes of this common procedure further. We aim to recognize, discuss, and propose a classification for HVI, considering the concept of the CORA.

## Materials and methods

Study method

We performed a retrospective observational radiographic analysis at the Trauma and Orthopedics Centre, Colchester Hospital, Colchester. We had the aim of studying and evaluating the HIA and the HIA-CORA in adult patients to assess interphalangeal deformity characteristics and measurement reliability. We collected radiographs of the foot from patients who attended the hospital between August 2017 and September 2022 for "bunion" or "big toe deformities." Radiographs with apparent rotational deformity were also mitigated as best as possible.

Inclusion Criteria

Inclusion criteria include patients over 18 years of age, both males and females, who had anterior-posterior (AP) view radiographs of their weight-bearing feet and presented to the hospital in an elective setting. 

Exclusion Criteria

Radiographs where a sagittal plane (flexion) deformity was seen on the lateral radiograph to reduce confounding factors; non-weight-bearing radiographs of the foot; and radiographs with foreign bodies/trauma to the foot. 

We did not necessarily include normal, asymptomatic feet in our study. A sample size was assembled for patients who had visited the Trauma and Orthopedics Center at Colchester Hospital for the duration of August 2017 to September 2022. After applying the inclusion and exclusion criteria, we analyzed a total of 80 radiographs for analysis. The final sample size was determined by the feasibility and data availability during the study period. We did not perform a formal "priori" sample size calculation, but a post hoc power analysis did confirm that a minimum of 65 radiographs would suffice to provide >90% power to detect a large effect (w=0.5) in the distribution of CORA types using a chi-square test with an alpha of 0.05. Thus, a sample dataset of 80 radiographs seemed adequate for our study. 

Radiographs

A total of 80 radiographs were collected from the patients included in this study. Each X-ray was anonymized and given a numerical identifier to protect confidentiality. The upper limit of the HIA was deemed to be 10 degrees of valgus [2].

All the radiographs were exported to imaging software (Insignia Medical Systems Insight, Picture Archiving and Communication System (PACS) Insight Zero, Insignia Medical Systems, Hampshire, UK) for analysis.

Statistical analysis and interobserver reliability

Three independent observers completed the radiograph reviews to allow a study of intra- and extra-observer error to be considered. The HIA was measured for each X-ray. The HIA-CORA was determined after measuring angles at five controlled zones of the proximal phalanx and distal phalanx. 

We assessed the interobserver reliability for the continuous variable HIA using a two-way random interclass correlation coefficient (ICC). For categorical variables, i.e., CORA location/classification, we used Fleiss' Kappa (κ). 

## Results

The percentage of left foot X-rays was 22.5% (n=18), and the number of right foot X-rays was 77.5% (n=62). The male-to-female distribution was 31.25% (n=25) male and 68.75% (n=55) female. 

The points of reference were as follows: P1=basal metaphysis of the proximal phalanx, P2=midpoint of the proximal phalanx, P3=distal metaphysis of the proximal phalanx, P4=basal metaphysis of the distal phalanx, and P5=interphalangeal joint (Table 1).

**Table 1:** Centre of rotational angulation locations

Centre of Rotational Angulation	P1- Base of Proximal Phalanx	P2- Midpoint of the Proximal Phalanx	P3- Distal End of the Proximal Phalanx	P4- Base of the Distal Phalanx	P5- Interphalangeal Joint	No Hallux Interphalangeus	Total
N	7	10	20	6	4	18	65

Radiographic findings indicated that the prevalence of “normal” hallux in the population was 27.69% (n=18). The percentage of feet with HVI was 72.30% (n=47). Among those with HVI, it was noted that the commonest deformity, with 30.76% (n=20), presented with the CORA at the distal end of the proximal phalanx (P3). The second most prevalent type of HVI is with the CORA at the midpoint of the proximal phalanx (P2) (15.3%) (n=10). The remaining classes had a CORA based at the proximal end of the proximal phalanx (P1) at 10.7% (n=7), a CORA at the base of the distal phalanx (P4) (9.23%) (n=6), or a CORA within the distal interphalangeal joint (P5) (6.15%) (n=4).

When the CORA is based at the base of the proximal phalanx (P1), we get a mean of 13.35° ± 1° (range 11.5°-14.9°) of deformity. When the CORA is based at the midpoint of the proximal phalanx (P2), we get a mean of °± (range 10.3°-14.1°) of deformity. When the CORA is based at the distal end of the proximal phalanx (P3), we get a mean of °± (range 10.3°-20.5°) of deformity. When the CORA is based at the base of the distal phalanx (P4) we get a mean of °± of (range 9.8°-24°) deformity. When the CORA is based at the interphalangeal joint (P5), we get a mean of 12.44°± (range 9.1°-15.1°) of deformity.

The interobserver reliability assessment for the HIA measurements demonstrated significant agreement between the observers, with the ICC being 0.92 (95% confidence interval=0.88-0.95; P<0.001). The agreement for classifying the CORA location was substantial, with a Fleiss' Kappa (κ) of 0.78. Any discrepancies in CORA classification were resolved by consensus amongst the three observers, and these consensus values were used for all subsequent analyses.

## Discussion

Overview

Hallux interphalangeus (also known as HVI) has a multifactorial etiology. Early descriptions (Daw, 1935) defined HVI as >10° lateral deviation at the interphalangeal joint (IPJ) [2,4]. Proposed causes include congenital and developmental factors such as growth-plate abnormalities of the phalanges [5,6]. For example, disturbed growth of the phalangeal condyles or distal phalangeal base can yield an angulated IPJ [6]. Biomechanical factors are also implicated: chronic lateral pressure or constrictive footwear in youth may impede lateral development of the distal phalanx, increasing HIA [7]. Recent data show HVI is far more common in juvenile-onset hallux valgus than adult-onset, suggesting developing feet with ongoing growth are especially susceptible to these influences [7,8].

Other factors include generalized ligamentous laxity, first-ray malalignment, and genetic predisposition (as in familial bunion deformities). In many cases, however, an exact trigger is not identified. Importantly, traumatic injury is uncommon; Grawe et al. found pediatric HVI cases had no history of trauma [9]. Finally, accessory ossicles or exostoses (e.g., a large lateral ossicle, as reported by Primadhi et al.) can be associated with HVI, either developmentally or post-traumatically [5].

The excellent interobserver reliability for HIA measurements (ICC=0.92) and the substantial agreement for CORA localization (κ=0.78) confirm that this classification system is reproducible and can be consistently applied by different observers. This aligns with previous studies by Khademi et al. and Strydom et al., which also found standardized radiographic measurements of the hallux to be reliable [2,10].

In our study, 72.3% of the radiographs of patients who presented to the hospital and underwent radiographs of the foot exhibited HVI, which is consistent with earlier population-based studies [2,9]. The majority of the deformities were centered around the distal metaphysis of the proximal phalanx, which supports the hypothesis that asymmetrical growth at/near the proximal phalanx physis may be a common etiologic pathway. 

Diagnosis of HVI 

Clinical Findings

Clinically, HVI manifests as a bent great toe at the IPJ, often with a palpable medial prominence or bursa on the toe. Patients may report toe pain or difficulty fitting shoes if the deformity is pronounced. Examination may reveal a valgus angulation of the distal phalanx (in the coronal plane), while the metatarsophalangeal (MTP) joint may be normal or also involved in hallux valgus. A tight lateral capsule or constricted medial toe soft tissues can be present. In some cases (especially when HVI is mild or asymptomatic), no pain occurs, and the issue is noted incidentally on imaging or as a cosmetic concern. Notably, Grawe et al. observed that symptomatic pediatric HVI often had a distinct bony exostosis at the medial IPJ; excision of this exostosis with soft-tissue realignment relieved symptoms [9]. In contrast, pain-free deformities that do not impair function may be managed non-operatively [5,9].

Imaging Findings

Radiographic evaluation is essential. Weight-bearing AP foot views are standard, with the HIA measured by bisecting the shafts of the proximal and distal phalanges [3,10]. An HIA >10° typically defines HVI. Nguyen et al. reported a mean HIA of 13.5° in their cohort (range ~1-24°) [11], confirming that “mild” HVI (10-15°) is common. In that study, 78% of feet had an HIA >10° [11]. Higher values (>20°) are less frequent (about 10% of cases) but often symptomatic [11]. Clinicians should also assess the distal phalanx-MTP joint angle (F2-MTP), distal articular set angle (DASA), and IP obliquity.

Standard AP views may underestimate the true HIA in some deformities. A specialized “off-axis” oblique view has been described to better profile the IPJ [12]. Kaufmann et al. found that traditional AP radiographs undershot the HIA by up to ~5°, whereas the off-axis view yielded larger angles and higher interobserver reliability [12]. Thus, when the clinical suspicion is high, obtaining an off-axis (slightly rotated or toe-up) view can improve accuracy. Advanced imaging (e.g., weight-bearing CT) is seldom needed for HVI alone but may be used in complex or revision cases.

Measurement reliability is generally good: Khademi et al. found inter- and intra-observer agreement for HIA within 5° in over 80% of readings [10]. Radiologists often report HIA alongside hallux valgus angle (HVA) and intermetatarsal angle (IMA) on foot films to document any IP component of deformity. In summary, diagnosis of HVI relies on a combination of clinical assessment (toe alignment, pain) and confirmed radiographic angle >10° [1,10]. 

Theoretical rationale for the CORA-based classification

The fundamental principle of the CORA, well-established in long-bone deformity correction, is that the most efficient and anatomical correction occurs at the apex of the deformity [13]. Our classification applies this principle to the phalanges of the hallux. The high prevalence of deformities centered at the metaphyseal regions (Types I, III, and IV) may suggest a developmental origin related to asymmetric physeal growth, a hypothesis supported by existing literature [5,6]. However, this remains a theoretical inference from our radiographic data and requires further etiological investigation. 

The value of this classification lies in its potential to provide a structured, anatomical language for describing HVI. Moving beyond a single angular measurement (HIA) to define the location of the deformity, it offers a more nuanced understanding of the patho-anatomy.

Within the population of normal HVA (less than or equal to 10 degrees of valgus), it was not possible to determine a single point of CORA with reproducibility.

Whilst of interest when considering more holistic growth staging problems, it is in the management of the pathological HVI surgically that adjustments to practice can be inferred from this. The current basal osteotomy has been described in multiple techniques, but is most commonly known as the ‘Akin.’ This closing wedge tricortical osteotomy principle is performed at varying angles based on experience and training, and here we propose a principle by which to use the CORA as a guide to technique, optimizing the point of correction. 

**Figure 1:**
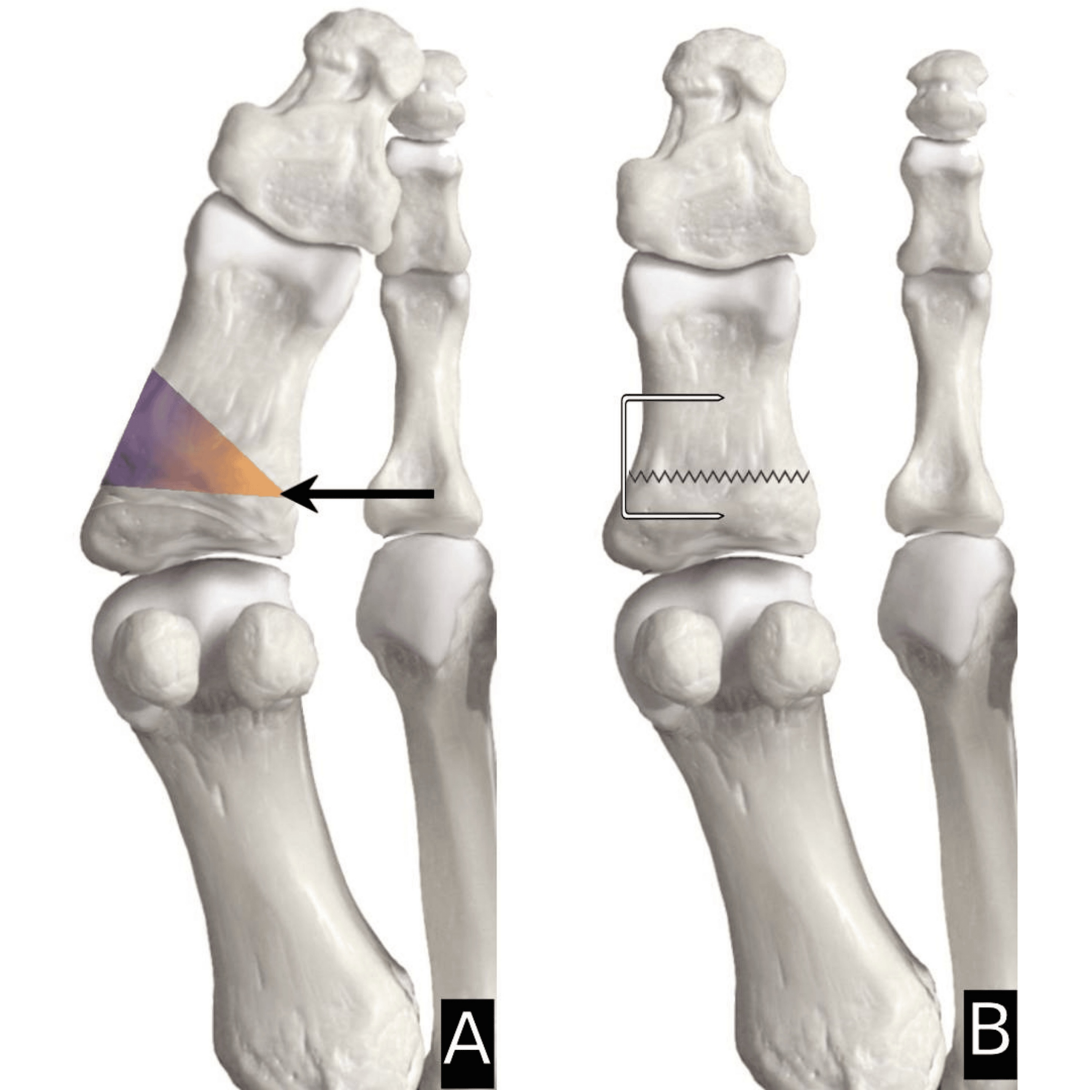
CORA at P1 with suggested procedure A: arrow indicating the center of rotation of angulation (CORA) at the basal metaphysis of the proximal phalanx (P1) and angulation caused in the shaded gradient; B: suggested procedure, which shows a corrective horizontal closing wedge in the proximal metaphysis secured with a staple. Image created by author Suvank Rout using Adobe Photoshop CC (Adobe Inc., CA, USA).

**Figure 2:**
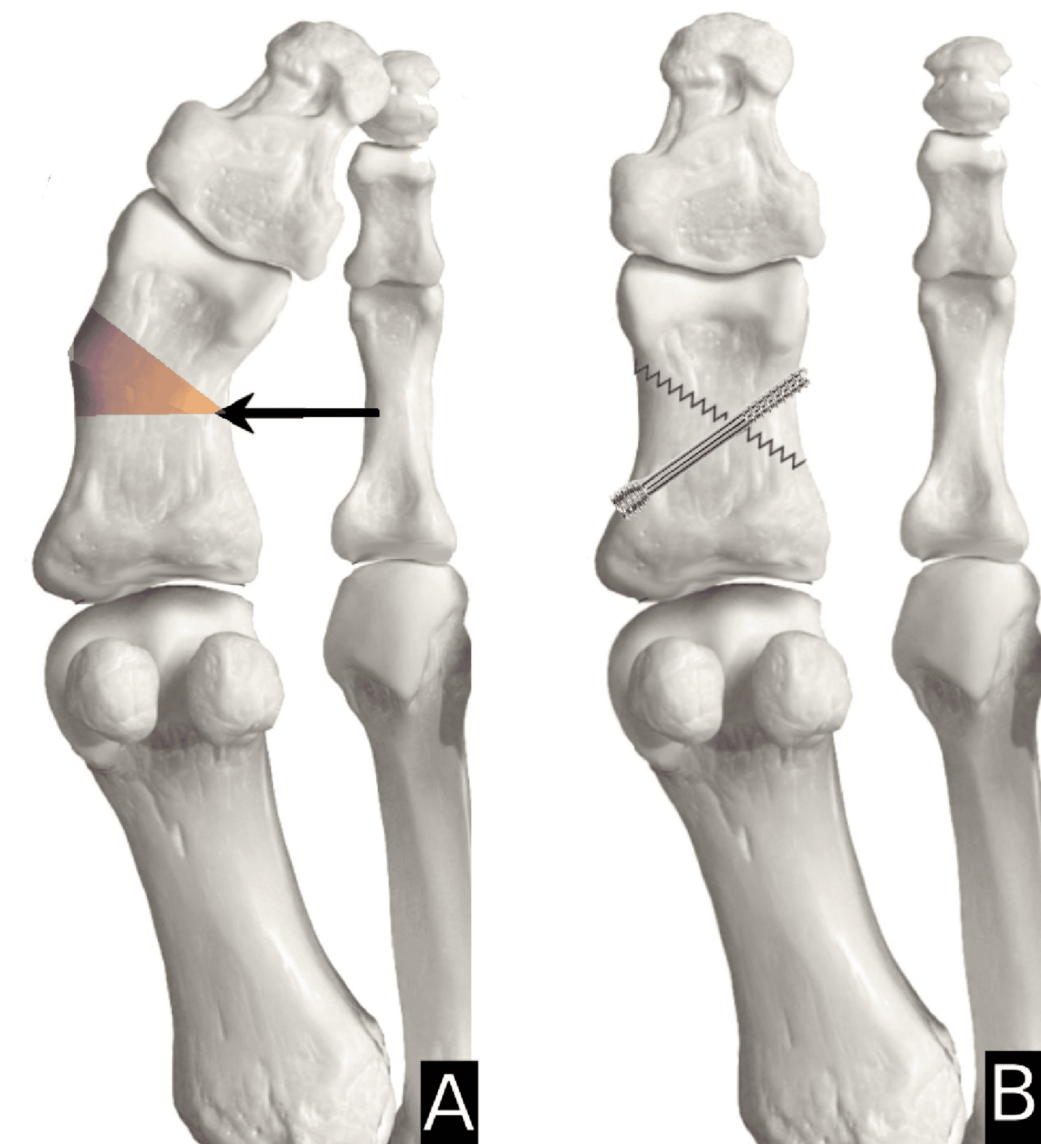
CORA at P2 with suggested procedure A: arrow indicating the center of rotation of angulation (CORA) at the midpoint of the proximal phalanx (P2) and angulation caused in the shaded gradient; B: suggested procedure that shows a corrective oblique closing wedge with a screw. Image created by author Suvank Rout using Adobe Photoshop CC (Adobe Inc., CA, USA).

**Figure 3:**
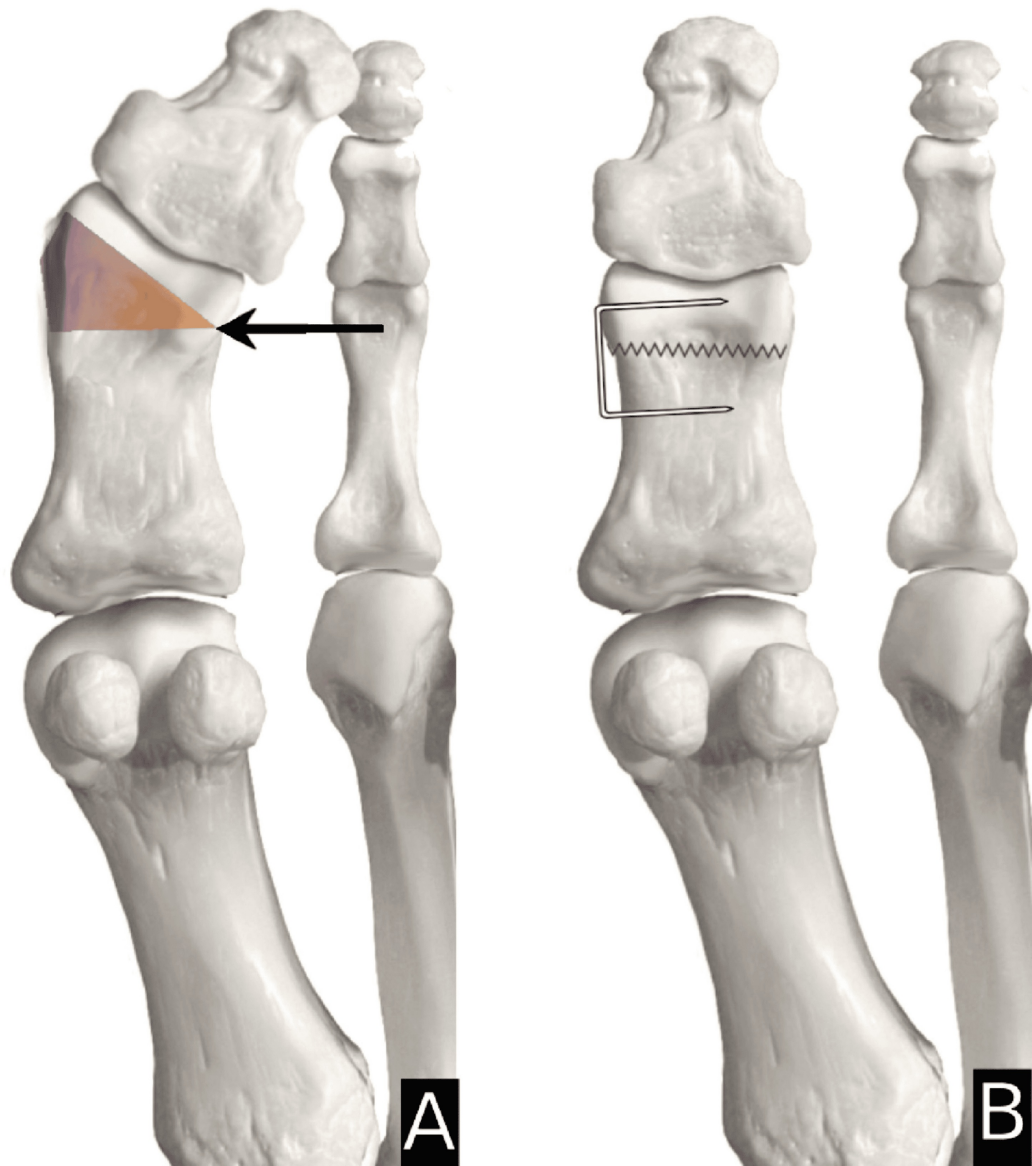
CORA at P3 with suggested procedure A: arrow indicating the center of rotation of angulation (CORA) at the distal metaphysis of the proximal phalanx (P3) and angulation caused in the shaded gradient; B: suggested procedure that shows a corrective horizontal closing wedge in the distal metaphysis secured with a staple. Image created by author Suvank Rout using Adobe Photoshop CC (Adobe Inc., CA, USA).

**Figure 4:**
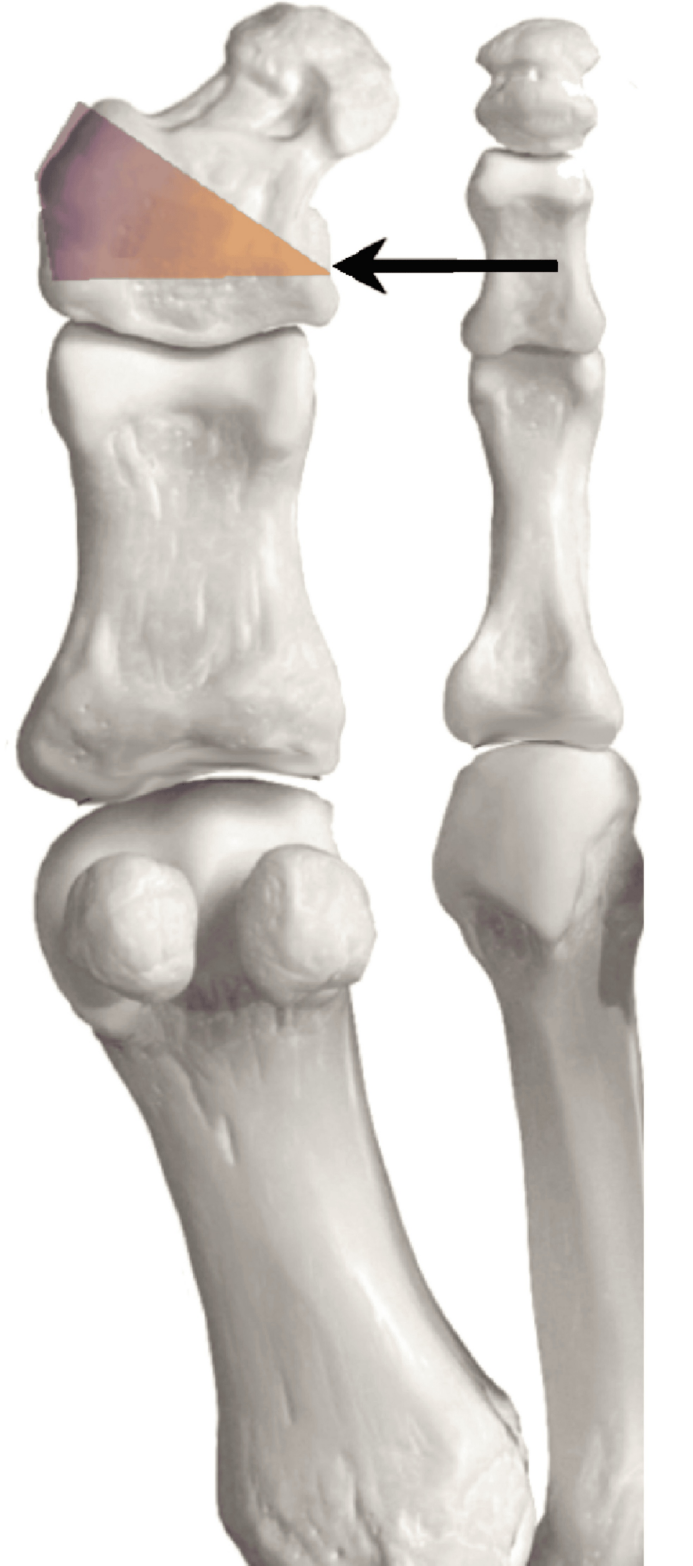
CORA at P4 with suggested procedure Arrow indicating the center of rotation of angulation (CORA) at the basal metaphysis of the distal phalanx (P4) and angulation caused in the shaded gradient. Image created by author Suvank Rout using Adobe Photoshop CC (Adobe Inc., CA, USA).

**Figure 5:**
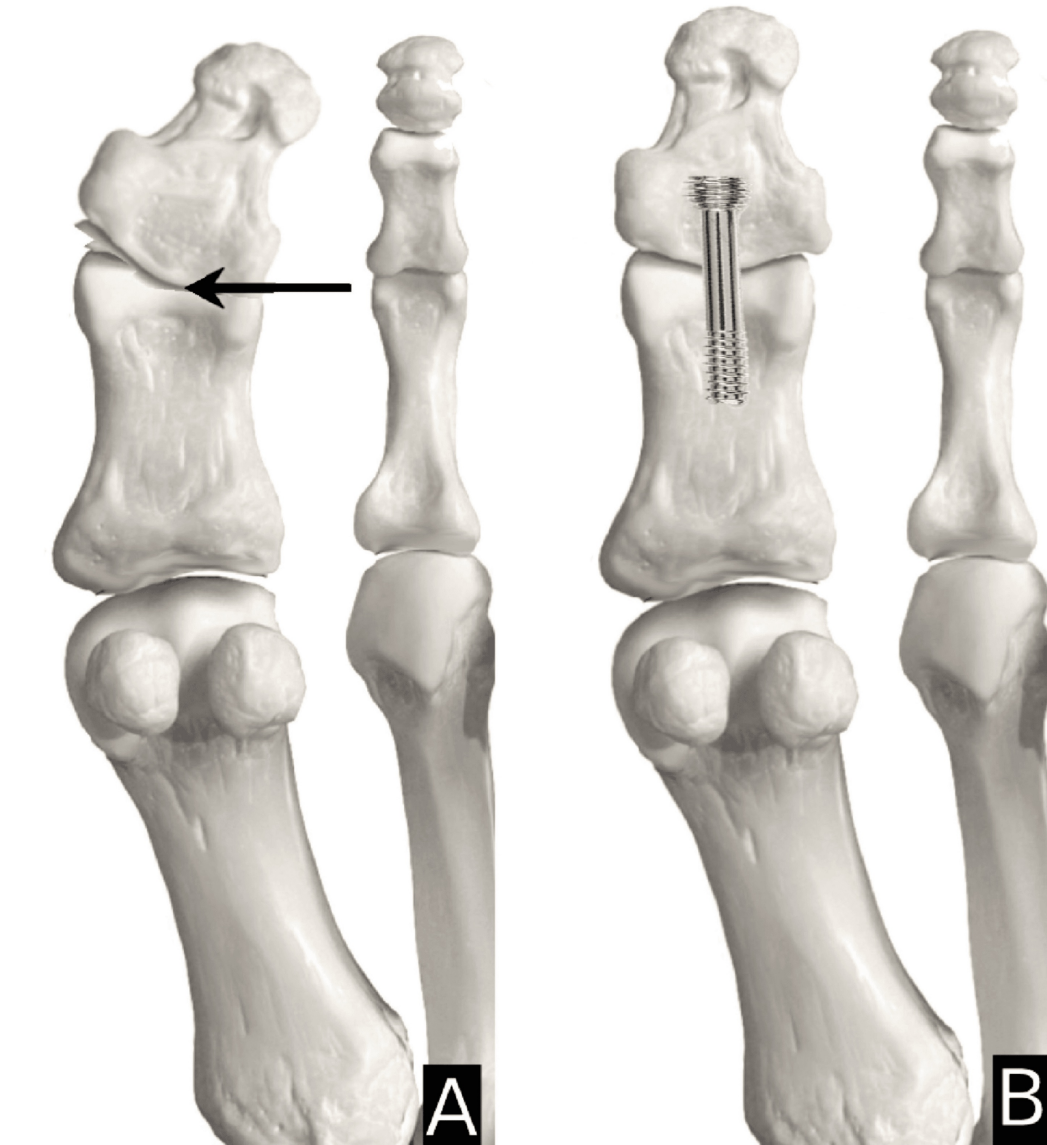
CORA at P5 with suggested procedure A: arrow indicating the center of rotation of angulation (CORA) at the interphalangeal joint (P5) and angulation; B: suggested procedure that shows corrective oblique interphalangeal joint fusion. Image created by author Suvank Rout using Adobe Photoshop CC (Adobe Inc., CA, USA).

Type 1=horizontal closing wedge in proximal metaphysis secured with staple (Figure 1); Type 2=oblique closing wedge secured with screw (Figure 2); Type 3=horizontal closing wedge in distal metaphysis secured with staple (Figure 3); Type 4=cannot correct at CORA, cautious proximal phalanx wedge correction (Figure 4); and Type 5=corrective IPJ fusion (Figure 5). 

The introduction of a CORA-based classification for HVI provides a structured and anatomically logical framework for both assessment and surgical correction, which are described in Table 2. Based on the radiographic apex of angulation, five deformity types are proposed. Table 1 provides a clinical pathway to link the radiological findings and surgical techniques, which could potentially improve preoperative planning and reduce the risk of recurrence or under-correction. 

**Table 2:** Proposed classification, along with the anatomical description and suggested surgical implications Literature supporting the surgical implication/preferred correction has been cited in the table.

Type	Centre of Rotational Axis Location/Zone	Anatomical Description	Surgical Implication/Preferred Correction
Type I	P1 – Base of proximal phalanx	Basal metaphyseal deformity, close to the Akin osteotomy site	Proximal Akin osteotomy or closing wedge osteotomy fixed with a staple or screw [2,13,14]
Type II	P2 – Midpoint of proximal phalanx	Mid-diaphyseal deviation	Modified Akin or wedge osteotomy; may be percutaneous [6,11,15]
Type III	P3 – Distal metaphysis of proximal phalanx	Most common type: deformity apex distal to standard Akin osteotomy	Distal Akin osteotomy or Vander Griend technique for direct correction at P3 [15,16]
Type IV	P4 – Base of distal phalanx	Deformity close to the interphalangeal joint; may present with ossicle formation or exostosis	Distal phalangeal wedge osteotomy or exostectomy; may require percutaneous approach [5,17]
Type V	P5 – Within the interphalangeal joint	True intra-articular deformity; often associated with arthritic changes	Interphalangeal joint arthrodesis (crossed screw technique) or fusion [6,18]

Potential clinical implications

The application of CORA-based analysis to the hallux provides surgeons with a geometric rationale for osteotomy planning, similar to that used in long-bone deformity correction. This method ensures correction occurs at the true apex of deformity, reducing the risk of creating secondary translational malalignment. Furthermore, this stratification enhances the precision of preoperative planning and supports reproducibility across surgeons.

This classification system thus allows surgeons to be able to localize the deformity apex precisely and be able to plan the corrective osteotomy or suggested procedures at the true CORA, thus minimizing translation errors and helping to preserve the bone stock of the patient. Neuman et al. and Eldessouky et al. have demonstrated in their study that modern fixation techniques, such as low-profile staples or screws, can achieve stable correction with minimal complications when the osteotomy is aligned with the actual deformity apex [13,14]. 

The patient-perceived benefit of HVI correction is to reduce the consequences of the excessive deviation, including hard and soft corns over promontories, crowding of the lesser toes, and medial callus from abnormally sited gait-related load [19]. However, the surgeon must look to the IPJ alignment when correcting this, both because a 3D-aligned IPJ will likely optimize symptom relief and because an overcorrected osteotomy causing a varus deformity will set up consequences in years to come. 

Type 4, the recognition of an HVI in which the deformity is distal to the IPJ, must alert the surgeon, as mentioned. It is imperative to maintain or improve the IPJ axis to be perpendicular to the pull of both the flexor and extensor hallucis tendons. An osteotomy in the distal phalanx is ill-advised owing to the technical difficulties of nail bed origin, etc. Any necessary corrective osteotomy of the proximal phalanx must therefore be carefully considered to balance improvement over consequence from the IPJ.

The Type 3 (P3) deformity was the prevalent deformity in the study cohort and is especially suited to a distal Akin osteotomy, as first described by Vander Griend [16]. Yañez Arauz et al. demonstrated that percutaneous osteotomy of the distal phalanx effectively corrects F2-IP angles (11.7° to 2.4°) without complications [6]. Distal deformities such as Type 4 (P4) or Type 5 (P5) may benefit from percutaneous phalangeal osteotomy [6] or arthrodesis where intra-articular destruction is present [18]. The reliability of our measurement approach (ICC 0.92) aligns with Khademi et al. and Hujazi et al., confirming that reproducibility is achievable when observers adhere to standardized bisection methods [10,20]. A recent study by Hujazi et al. showed that angular measurements of HIA have excellent intra- and interobserver reliability for both pre- and post-hallux valgus surgery [20].

Limitations of the study 

The retrospective design of this study can introduce a selection bias, such as the sample comprising radiographs from patients presenting for orthopedic appointments. As this study did not focus on any clinical domains, this limits conclusions regarding functional severity. 

We state that these are hypotheses derived from radiographic correlation, not conclusions supported by clinical outcomes data. This classification is presented as a tool for future research and academic discussion, not as a validated clinical guideline. Therefore, validation is the critical next step. Future studies must be prospective and correlate CORA type with patient-reported symptoms, functional limitations, and surgical outcomes. This article does not focus on potential rotational deformities; however, none of the surgical techniques proposed preclude the toe during an Akin procedure, as would be standard in current practice. There are also other implants by which the osteotomies can be secured that are out of this review. As only AP-view radiographs were analyzed in this study, this may limit visualization. Use of 3D imaging or off-axis projections could improve deformity visualization, as noted by Kaufmann et al. [12]. Irrespective of these limitations, due to the high interobserver reliability, we were able to validate the CORA-based approach.

Future directions

Future studies should validate this classification in a larger, prospective, and multicentre cohort. Studies should correlate the CORA type with surgical outcomes and recurrence rates, and dynamic load distribution patterns using pedobarography. 

## Conclusions

This study proposes and validates a reproducible, CORA-based radiographic classification for HVI. In our studies, we noted that the most common type of CORA in HVI was the P3 type, where the CORA was at the distal end of the proximal phalanx (43%). The second most common type of CORA from our cohort was noted to be the midpoint of the proximal phalanx (21%). In conclusion, this study proposes a reproducible, CORA-based classification system for HVI that identifies five predominant deformity patterns. The high interobserver reliability confirms its utility as a radiographic descriptive tool. While this system provides a novel anatomical framework and a theoretical basis for refining surgical strategy, its clinical efficacy and impact on patient outcomes remain to be proven. We present this classification as a foundation for future prospective clinical research to determine its true value in the management of HVI.
